# Fatal Pulmonary Embolism in the Setting of Immune Reconstitution Inflammatory Syndrome Attributed to Ovarian Tuberculosis

**DOI:** 10.1177/2324709617729690

**Published:** 2017-09-08

**Authors:** Israel Ugalde, Daniela Pirela, Saberio Lo Presti, Molly Bilderback, Andrés Pirela, Joseph Chan

**Affiliations:** 1 Mount Sinai Medical Center, Miami Beach, FL, USA; 2 La Universidad del Zulia, Maracaibo, Venezuela

**Keywords:** *Mycobacterium tuberculosis*, immune reconstitution inflammatory syndrome, human immunodeficiency virus, tubo-ovarian abscess, necrotizing granulomata

## Abstract

In developed countries, tuberculosis remains a health care challenge due to human immunodeficiency virus (HIV) and immigration from endemic regions. The Centers for Disease Control and Prevention reported 9557 new cases in 2015, with extrapulmonary involvement in 20.2% of the cases. We present a 33-year-old woman from Cape Town, South Africa, who developed abdominal pain and fever while working on a cruise ship. She sought medical where she underwent computed tomography of her chest, abdomen, and pelvis with findings suggestive of pulmonary tuberculosis and an 8.9-cm pelvic mass. HIV testing was positive and the patient was started on antiretroviral therapy. Bronchoscopy confirmed the presence of acid-fast bacilli, and she was started on rifampin, isoniazid, pyrazinamide, and ethambutol. She remained persistently febrile, raising suspicion for immune reconstitution inflammatory syndrome. However, despite empiric antibiotics, the patient remained persistently febrile, tachycardic, and on day 10 of admission she went into ventricular fibrillation and expired. Autopsy revealed an occlusive thrombus in the left main pulmonary artery in addition to necrotizing granulomata in multiple organs and bilateral tubo-ovarian abscesses. Postmortem cultures for were positive for *Mycobacterium tuberculosis*, all consistent with disseminated *Mycobacterium tuberculosis*. Although previous reports underscore the association between tuberculosis and hypercoagulability, the exact mechanism remains unknown. In this article, we report a case of disseminated tuberculosis complicated by bilateral tubo-ovarian abscesses with fatal pulmonary thrombus formation.

## Introduction

In developed countries, tuberculosis (TB) remains a health care challenge due to human immunodeficiency virus (HIV) and immigration from endemic regions. Worldwide, 10% to 15% of all HIV-related deaths are associated with TB, and it is considered a leading cause of death. Even in the Unites States, the Centers for Disease Control and Prevention reported 9557 new cases in 2015. Extrapulmonary involvement was responsible for 20.2% of the new cases with genitourinary TB only accounting for 91 cases (4.5%).^1,2^

Tuberculosis has been shown to improve the ability of HIV to replicate by activating CD4 T-lymphocyte cells and macrophages. The prolonged increase in HIV replication can accelerate disease progression and induce a pro-inflammatory state.^3^ The pro-inflammatory state increases the risk for inappropriate immune system activation leading to immune reconstitution inflammatory syndrome (IRIS).^4^ IRIS is the paradoxical worsening of an opportunistic infection or neoplasm when an HIV-positive individual is started on antiretroviral therapy (ART).^4^

In this article, we report a particularly interesting case of a patient with recently diagnosed HIV who was found to have bilateral adnexal masses as a presentation of disseminated TB, who unfortunately died from a massive pulmonary embolism in the setting of IRIS-TB syndrome.

## Case Presentation

This was a 33-year-old African woman from Cape Town, South Africa, who developed abdominal pain and fever while working on a cruise ship. While on the cruise, she developed abdominal pain for 3 days. Initially she was treated for constipation but sought out medical care at the next port when she reported new-onset subjective fevers. She never experienced a cough, chest pain, or shortness of breath with the fever and abdominal pain. The patient usually traveled from Cape Town to Texas and then to different islands of the Caribbean. Four weeks prior to presentation she was vacationing with her family in South Africa for about 7 weeks, where she noticed appetite loss and weight loss, but she thought this was normal for her before leaving for another cruise trip.

On arrival to the initial medical center, computed tomography (CT) imaging showed a left upper lobe consolidation concerning for TB as well as a pelvic mass. HIV testing came back positive along with a CD4 count of 114 cells/mm^3^. She was started on darunavir, emtricitabine-tenofovir, and ritonavir for ART and then transferred to the referral center for a bronchoscopy.

At the referral center, the patient was febrile but hemodynamically stable and comfortable in bed. On arrival she underwent bronchoscopy to obtain bronchoalveolar lavage samples, which later on confirmed the presence of acid-fast bacilli, leading to immediate initiation of rifampin, isoniazid, pyrazinamide, and ethambutol (RIPE). Despite RIPE therapy, the following day the patient continue spiking fevers up to 106.5°F, raising the suspicion for IRIS. ART was discontinued after 1 week of therapy in an attempt to resolve persistent fevers and overall clinical deterioration.

**Figure 1.**
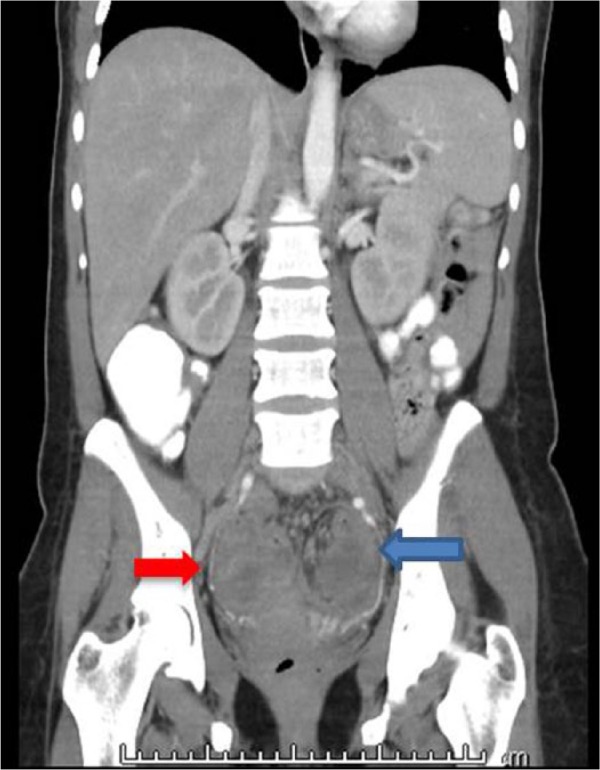
CT of the abdomen with contrast, coronal view.

**Figure 2.**
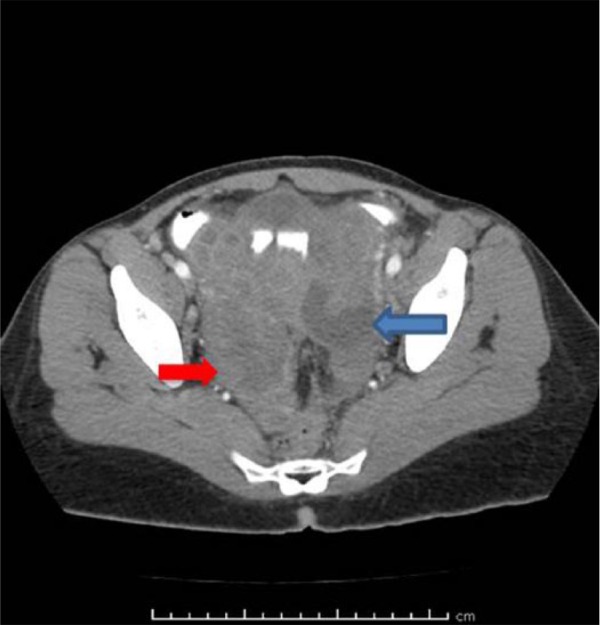
CT of the abdomen with contrast, transverse view: large hypodense bilateral pelvic mass.

During the hospitalization, a pelvic ultrasound confirmed the bilateral adnexal masses up to 8.9 cm with internal vascularity, and empiric antibiotics were started for a possible tubo-ovarian abscess (TOA; Figures 1 and 2). Urine nucleic acid amplification testing for chlamydia and gonorrhea were negative, and CA-125 tumor marker was found to be elevated 5 times above the upper limit of normal. The patient underwent a biopsy of the mass along with CT-guided catheter drainage of the fluid-filled abscesses with interventional radiology. The biopsy did not yield tissue, and instead, 25 mL of purulent fluid was obtained from the left adnexal mass.

**Figure 3.**
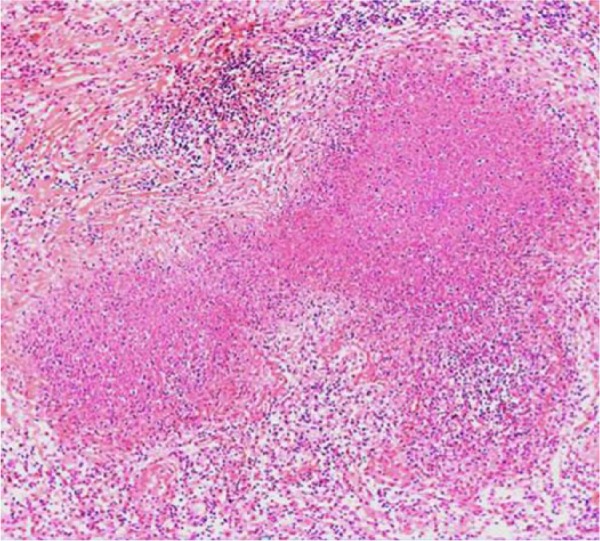
Hematoxylin-eosin stain of the ovarian tumor mass showing suppurative necrotizing granuloma.

**Figure 4.**
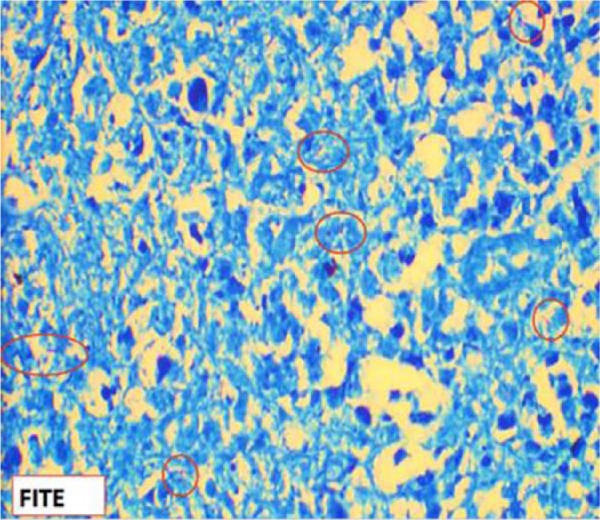
Fite stain of the ovarian mass with evidence of acid fast bacilli.

Despite antibiotic therapy and drainage of the abscesses, the patient remained persistently febrile and tachycardic. Suddenly on day 10 of admission, she developed ventricular fibrillation and became unresponsive. The advanced cardiovascular life support protocol was quickly initiated but the patient never regained a pulse and was pronounced deceased. An autopsy was performed and concluded that a 3 cm by 0.5 cm occluding thrombus in the left main pulmonary artery was the main cause of death. In addition, the patient was found to have necrotizing granulomata of the lungs, omentum, pancreas, gallbladder, liver, spleen, bone marrow, periaortic and pelvic lymph nodes, as well as bilateral TOA with postmortem cultures positive for *Mycobacterium tuberculosis* (Figures 3 and 4). State laboratory testing concluded the patient had pan-sensitive TB to RIPE therapy as well as a negative Hain test for suspected mutations that can lead to multidrug-resistant TB. All findings were consistent with disseminated TB.

## Discussion

Tuberculosis is one of the top 10 causes of death worldwide and the leading killer of HIV-positive population. South Africa is 1 of the 6 countries that account for 60% of total TB cases worldwide.^5^ IRIS due to TB, also called TB-IRIS, is characterized by transient but severe localized or systemic inflammatory reactions against *Mycobacterium tuberculosis* antigens. Common signs and symptoms include fever, hepatitis, increased intracranial pressure, uveitis, sarcoidosis, and pulmonary infiltrates. The pathophysiology of TB-IRIS is largely speculative but believed to be a type IV hypersensitivity reaction.^4^ The lymphocyte CD4-Th1 immune cells increase production of pro-inflammatory cytokines interleukin-6 (IL-6), tumor necrosis factor-α (TNF-α), and in particular, interferon-γ (IFN-γ).^6^ These cytokines are known to lead to a widespread neutrophilic response.^4^ Other CD4 T-helper cells, such as Th17 cells, which are characterized by their production of IL-17, have been suggested to play a part in TB-IRIS but their role has not been well described.^6^

In order to reduce the chance of developing IRIS, guidelines recommend that ART-naïve patients with CD4 counts ≥50 cells/mm^3^ initiate ART usually in 2 to 4 weeks, but within 8 weeks, of starting TB treatment.^7^ On the other hand, the benefits of immediate ART, within 2 weeks, are believed to outweigh the risk of developing IRIS in patients with CD4 counts <50 cells/mm^3^. In terms of treatment, IRIS is usually transient, and mild to moderate cases can be symptomatically treated with nonsteroidal anti-inflammatory agents. For more severe cases, it is recommended to add steroid therapy. If a patient develops IRIS, it is recommended that RIPE and ART therapy be continued for the overall long-term health of the patient. Optimal dosing, duration, and efficacy of steroids have not been established and are based mostly on expert opinion.^7^ Due to the overall clinical deterioration of the patient, the decision was made to discontinue ART. Despite our best efforts, the patient developed ventricular fibrillation and expired on hospital day 10. Interestingly, the autopsy concluded pulmonary embolism as the presumable cause of death and confirmed the presence of disseminated TB.

Genitourinary TB is the second most common site for extrapulmonary TB.^8^ Isolated ovarian TB is an uncommon disease given that genitourinary TB usually presents with endometrial and fallopian tube involvement.^8^ Initial diagnoses of TOA or ovarian malignancy can be mistaken given the similarities in clinical, radiologic, and serum markers such as an elevated cancer antigen-125 (CA-125).^8-12^ CA-125 is elevated in 80% of postmenopausal ovarian carcinomas.^8^ However in premenopausal women, elevations in CA-125 have been noted in endometriosis, TB, and other nonneoplastic diseases.^8^ This makes CA-125 relatively nonspecific and nondiagnostic in premenopausal women. A thorough family history may narrow the differential diagnosis from an infectious etiology, such as TB in patients with multiple family members who had TB, to malignancy in a family history of ovarian cancer.^10^ However, tissue sample in multiple case reports have proven to be essential in aiding the history and physical exam.^8^

There are case reports in the literature associating hypercoagulability with *Mycobacterium tuberculosis* infection: deep vein thrombosis associated with pulmonary TB, peritoneal TB with secondary portal vein thrombosis, and fatal massive pulmonary embolism despite receiving appropriate RIPE therapy and a therapeutic international normalized ratio.^13-15^ This is the first reported case, to the authors’ knowledge, of death from a pulmonary embolism in a patient with TB-IRIS from disseminated genitourinary TB.

The hypercoagulable state seen in TB infection is due to the imbalance of procoagulants and anticoagulants. These are represented by elevated levels of thrombin-antithrombin complexes, D-dimer, and fibrinogen along with reduced levels of antithrombin, protein C and S.^2^ When immune cells come in contact with TB there are increased amounts of TNF-α, IL-1, and IL-6.^2^ This results in systemic inflammation. No predominant link between pulmonary TB and pulmonary embolism has been established favoring the concept of a systemic inflammatory state leading to hypercoagulability over local thrombosis.^2^ Database analysis has shown that TB is an independent risk factor of venous thromboembolism, especially in black patients. Hospital mortality increases in patients with TB and venous thromboembolism from 2.7% to 15%.^16^ Tuberculosis treatment, more specifically rifampin, has also been associated with hypercoagulability.^13^

## Conclusion

This case is unique because it emphasizes the importance of considering ovarian TB in females with pelvic masses given the proper epidemiologic history. Ovarian TB has the possibility to be the initial presentation of disseminated TB in HIV patients, and the potential risk for IRIS should be considered when deciding to start ART. It is crucial to raise awareness of the hypercoagulable state associated with TB that can be worsened by IRIS. This case report demonstrates the potential fatal consequences in a patient with disseminated TB who develops further systemic inflammation with TB-IRIS syndrome that can be detected early if the clinician has the appropriate index of suspicion.
